# Clinical Trial to Evaluate Safety and Efficacy of Transdermal Electrical Stimulation on Visual Functions of Patients with Retinitis Pigmentosa

**DOI:** 10.1038/s41598-019-48158-5

**Published:** 2019-08-12

**Authors:** Gen Miura, Takeshi Sugawara, Yohei Kawasaki, Tomoaki Tatsumi, Tomohiro Nizawa, Takayuki Baba, Hideki Hanaoka, Shuichi Yamamoto

**Affiliations:** 1 Department of Ophthalmology and Visual Science, Chiba University Graduate School of Medicine, Chiba, Japan; 2 Clinical Research Centre, Chiba University Hospital, Chiba, Japan

**Keywords:** Medical research, Clinical trials

## Abstract

To evaluate the safety and efficacy of transdermal electrical stimulation (TdES) with skin electrodes on improving the visual functions of patients with retinitis pigmentosa (RP), twenty eyes of 10 patients with RP underwent TdES (10-ms biphasic pulses, 20 Hz, 30 min) 6 times at 2 week intervals. All patients were stimulated bilaterally with 1.0 mA pulses. The primary endpoint was safety, and the secondary endpoints were the changes in the best-corrected visual acuity (BCVA), visual fields determined by the Humphrey field analyzer (HFA) 10-2 and Goldmann perimetry, and answers to the Visual Function Questionnaire-25. All of the 10 enrolled patients completed the study according to the protocol. No adverse events related to the treatments were reported during the follow-up examinations. The mean BCVA and Early Treatment Diabetic Retinopathy Study visual acuity were significantly improved after the TdES (P = 0.0078 and P = 0.001, respectively). The mean deviation of the HFA 10-2 was also significantly improved (P = 0.0076). We conclude that TdES with skin electrode is a safe therapeutic option and should be considered as a treatment option for patients with RP.

## Introduction

In 1955, Brindley reported that applying an electric pulses to the eye produced a light sensation called a phosphene^1^. Then, Potts *et al*. reported that electrically-evoked potential changes could be recorded from the cranial surface at the same time as the phosphenes by electrical stimulation of the eye^2^. Thus, applications of electrical pulses to the eye could be used to investigate the origin of phosphenes and electric evoked responses (EERs) evoked by electrical stimulation. In the 1990’s, Galli-Resta *et al*. reported that spontaneous afferent electrical activity regulated the targeted cell death in the developing rat visual system^3^. The results also showed that short periods of low frequency electrical stimulation accelerated axonal regeneration of peripheral neurons by Al-Majed *et al*.^4^. Morimoto *et al*. reported that the survival rate of rat retinal ganglion cells (RGCs) after transection of the optic nerve was significantly higher in rats whose optic nerve was electrically stimulated than that of the untreated group^5^. Morimoto *et al*. also found that electrical stimulation had a neuroprotective effect on RGCs, and they developed a transcorneal electrical stimulation device (TES) that could be used in humans^6^. They used a contact lens type of electrode that is used to record electroretinograms (ERGs), and the electrical pulses were obtained from an electrical stimulation device and delivered through the electrodes embedded in the contact lens. They studied the effects of TES on its neuroprotective activity on optic nerve diseases. Thereafter, it was reported that TES can protect the retinal photoreceptors in animal models of RP of rats and rabbits^7,8^, and in clinical studies on RP patients, TES improved the visual acuity, visual fields, and ERGs after TES has also been reported^9^.

Previous TES studies used the contact lens type or TDL fiber type corneal electrodes, whereas the electrical stimulation device used in this study was a patch containing the electrode that is applied to the skin, and electric stimulation is delivered through the skin so that it is less invasion and can be done more easily. This transdermal electrical stimulation (TdES) was performed using prototype equipment developed jointly with the Mayo Co., Ltd. A search of Medline/PubMed did not extract any publications reporting on the use of transdermal electrical stimulation using skin electrode for patients with retinitis pigmentosa.

Thus, the purpose of this clinical trial was to verify the safety and efficacy of TdES using skin electrodes for patients with retinitis pigmentosa and evaluate the visual functions before and after the TdES.

## Results

The recruitment for this trial began in June 2017 and ended on October 2017, and patients were enrolled between August 22, 2017 and November 21, 2017. The end date for the last patient to complete all of the protocol was February 20, 2018. Twelve patients were screened, and two were excluded due to violations of the inclusion criteria (2). In the end, 20 eyes of 10 patients were included in the statistical analyses. The methods were carried out in accordance with the relevant guidelines and regulations. The demographic and clinical characteristics of the patients at the baseline and at 12 weeks are shown in Table 1. The mean central foveal thickness was 165.28 ± 45.2 µm at the baseline.

**Table 1:** Demographic and clinical characteristics at baseline and at 12 week.

Parameters	Baseline	12 week	P Value
Number of patients/eyes		10/20	
Age (y/o)		52.7 (31 to 68)	
Male/Female		6/4	
AD/AR/Sporadic		3/2/5	
logMAR VA	0.349 ± 0.05	0.290 ± 0.05	0.0078
ETDRS VA (letters)	31.3 ± 2.3	35.4 ± 2.3	0.001
MD value of HFA10-2 (dB)	−22.854 ± 2.1	−22.189 ± 2.1	0.0076
Central 4 points of HFA10-2 (dB)	22.3 ± 1.5	22.313 ± 1.5	0.98
Area of visual field with target I/4 of GP	2820 ± 512	3335 ± 512	0.126
NEI VFQ-25 Compo 9	53.46 ± 6.55	50.73 ± 6.55	0.09

Numerical values are the means ± standard error of the means.

Abbreviations: A.D.: autosomal dominant, A.R.: autosomal recessive.

### Safety of this trial

Abnormalities such as keratitis, dermatitis around the skin electrodes, inflammations of the anterior ocular segment, opacities of the optic media, abnormalities of the fundus, facial and trigeminal nerve, and nasal abnormalities were not observed. There were no significant differences in the systolic and diastolic blood pressure and intraocular pressure between the baseline and the final visit values. The TdES was completed in all 10 cases, and there was no problem regarding tolerability to the electrical stimulation. Throughout the trial period, no trouble with the electrical stimulation device was observed.

### Efficacy of this trial

**Figure 1:**
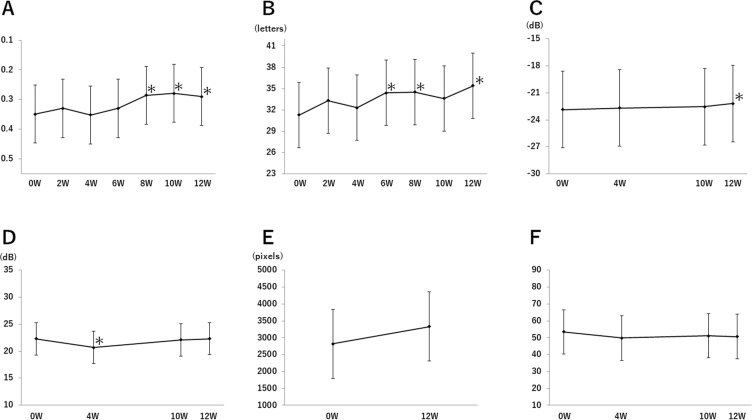
Changes in each parameter. (**A**) The change from 0 week in the mean VA in logMAR unit. (**B**) The change from 0 week in the mean ETDRS VA. (**C**) The change from baseline in the average of mean deviation of HFA 10-2. (**D**) The change from baseline in the mean value of the sensitivities of the central 4 points of HFA 10-2. (**E**) The change from baseline in the mean area of visual field with target I/4 of GP. (**F**) The change from baseline in the average of compo 9 of NEI VFQ-25. Means and their standard error were estimated by the linear mixed model. The asterisks indicate the significant changes from baseline. Whiskers indicated standard error.

The changes of each parameters from the baseline values (0 weeks) through the study period (12 weeks) are shown in Fig. 1. The mean BCVA was 0.349 at the baseline, 0.330 at 2 weeks, 0.352 at 4 weeks, 0.330 at 6 weeks, 0.286 at 8 weeks, 0.279 at 10 weeks, and 0.290 at 12 weeks (Fig. 1A). The BCVA was significantly improvement at 8 weeks (95% CI: −0.106–0.020, P = 0.0044), at 10 weeks (95% CI: −0.112–0.026, P = 0.0018), and at 12 weeks (95% CI: −0.102–0.016, P = 0.0078) compared with the value at the baseline.

The mean ETDRS BCVA was 31.3 at the baseline, 33.3 at 2 weeks, 32.3 at 4 weeks, 34.4 at 6 weeks, 34.5 at 8 weeks, 33.6 at 10 weeks, and 35.4 at 12 weeks (Fig. 1B). A significant improvement was observed at 6 weeks (95% CI: 0.7– 5.6, P = 0.0109), at 8 weeks (95% CI: 0.8–5.7, P = 0.0087), and at 12 weeks (95% CI: 1.7–6.5, P = 0.0010) compared with the value at the baseline.

The average mean deviation of the HFA 10-2 was −22.854 dB at the baseline, −22.674 dB at 4 weeks, −22.540 dB at 10 weeks, and −22.189 dB at 12 weeks (Fig. 1C). A significant improvement was observed at 12 weeks (95% CI: 0.184–1.147, P = 0.0076) compared to the value at the baseline.

The changes in the mean sensitivities of the central 4 points of HFA 10–2 are shown in Fig. 1D. The mean sensitivities of the central 4 points was 22.3 dB at the baseline, 20.663 dB at 4 weeks, 22.088 dB at 10 weeks, and 22.313 dB at 12 weeks. The decrease was significant at 4 weeks (95% CI: −2.945–0.330, P = 0.0150) compared to the baseline.

The changes in the mean area of visual field with target I/4 of GP are shown in Fig. 1E. The average of mean area was 2820.1 pixels at the baseline and 3334.9 pixels at 12 weeks (P = 0.1261; 95% CI: −158.7–1188.4).

The average of compo 9 of NEI VFQ-25 was 53.46 at the baseline, 49.82 at 4 weeks, 51.22 at 10 weeks, and 50.73 at 12 weeks (Fig. 1F). The decrease was significant at 4 weeks (P = 0.0268; 95% CI: −6.38–0.45) compared to the value at the baseline.

### Historical data of the patients

**Figure 2:**
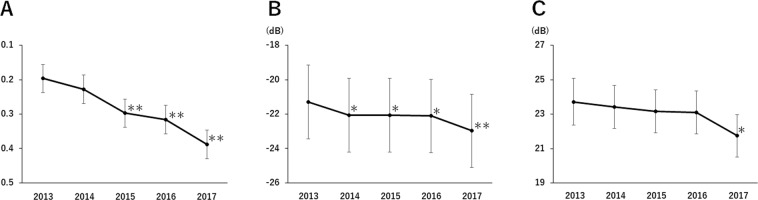
Historical data of the patients. (**A**) The change in the mean VA in logMAR unit for 5 years before this study. (**B**) The change in the average of mean deviation of HFA 10-2 for 5 years before this study. (**C**) The change in the mean value of the sensitivities of the central 4 points of HFA 10-2 for 5 years before this study. Means and their standard error were estimated by the linear mixed model. Whiskers indicated standard error. **P < 0.01, *P < 0.05.

Data of the all 10 patients for past 5 years before the clinical trial were shown in Fig. 2.

The changes of the BCVA in logMAR units (Fig. 2A), the mean deviation of HFA 10-2 (Fig. 2B), and the mean sensitivities of the central 4 points of HFA 10-2 (Fig. 2C) over the past 5 years were analyzed. BCVA in logMAR unit, the mean deviation of HFA 10-2 and the mean sensitivities of the central 4 points of HFA 10-2 were significantly worse in the past 5 years.

## Discussion

This study was the first clinical trial that assessed the safety and efficacy of transdermal electrical stimulation with skin electrode for patients with RP. It has been reported that TES using corneal electrodes improved the visual function of eyes with traumatic and ischemic optic neuropathy^10^, retinal artery occlusion^11^, and RP. However, corneal epithelial disorders such as dry eye occurred after the TES treatment^12^.

In our cohort, the electrical stimulation therapy was completed according to the protocol in all 10 patients. None of them dropped out due to irritation or pain from the electrical stimulation, and there was no problem on their tolerability to electrical stimulation. Throughout the trial period, no adverse events related to the corneal, skin, facial, and trigeminal nerves, and nasal disorders were found. We conclude that TdES is safe under our stimulating protocol.

Our results showed that the BCVA was significantly improved as has been reported. The mechanisms proposed for the improvement of visual functions include an increase in the level of IGF-1^6^, ciliary neurotrophic factor (CNTF) and brain-derived neurotrophic factor (BDNF)^13^, up regulation of Bcl-2 expression and down regulation of Bax expression^13^, increase in the chorioretinal blood flow^14^, inhibition of the NF-kB signaling pathway, and suppression of microglia activation^15^, up regulation of 25 proteins included cellular signaling proteins, proteins associated with neuronal transmission, metabolic proteins, immunological factors, and structural proteins^16^. Our results supported that these effects due to electrical stimulation contributed to improvement of visual function of RP patients.

A statistically significant improvement was observed from the 4th electrical stimulation session and at 6 weeks after the beginning of the TdES, and the improvement was maintained at 3 months. Bittner *et al*. reported significant improvements following each TES which prevented significant losses in the visual functions^17^. They also reported that the significant retinal blood flow improvements measured in the macular vessels showed after six weeks TES sessions^18^. Based on their results, improvement of visual function in our study may be related to retinal blood flow.

No significant change in the mean sensitivity of the central 4 points of HFA 10-2 was observed during this study period. A slow improvement was observed in the MD values of HFA 10-2, and statistically significant improvements were observed at 12 weeks. It has been reported that there was a significant increase in the amplitude of the photopic single flash b-wave and tendencies for an improvement for the scotopic b-wave amplitude in an earlier report after electrical stimulation in RP patients^12^. Our findings of an improvement in the MD values of HFA 10-2 are consistent with the previous report that the cone function was improved after TdES. One reason why there was not a significant improvement of the mean sensitivity of central 4 points was the high MD of 22.0 dB at the baseline.

Although there was no statistically significant change in the area of the GP, there was an increase in the number of pixels from 2883 pixels to 3222 pixels. This suggests that the peripheral visual field (rod degeneration) may have also improved by the TdES. This is consistent with the previous reports that the scotopic ERGs of rats and rabbits were improved by electrical stimulation^7,8,13^. Taking into consideration our HFA results, it is possible that TdES can be expected to improve the sensitivity of not only the central but also the peripheral field.

Recently, two groups reported that they did not detected significant improvements of the visual acuity and visual field after 1 year of transcorneal electrical stimulation in patients with retinitis pigmentosa^12,19^. They determined the stimulation current intensity based on individual electrical phosphene threshold. The values were lower than the current intensity used in this study. In addition, we stimulated both eyes at the same time, which was done because some of the current will flow to the opposite eye. Another reason is that because RP is mainly a binocular disorder and it is presumed that it will be a treatment with simultaneous stimulation of both eyes in the future. Therefore, it was necessary to conduct a study using a method that would actually. However, this means that there is a possibility that a more intense current larger than the current amount of 1 mA set in this study flows in one eye. These factors may be the reasons of the differences in the results of our findings from earlier studies.

The electrical stimulation did not lead to significant improvements in the VFQ score. Whether the QOL actually did not improve or whether VFQ was really appropriate as the evaluation method in this study, we need further study about correlation between electrical stimulation treatment and the quality of vision in RP patients.

There are several limitations in this study.

First, the number of RP patients was small and trial period was short. Bittner *et al*. reported that visual improvements after TES lasted for several months^17^. On the other hand, Schatz *et al*. reported no significant improvements were observed in the visual acuity and vision after one year^12^. In both of these studies and our study, there were differences in the electrode placements, level of current, and number of cases, and a simple comparison is difficult. However, the amount of current used in this study was stronger than that used in those studies. When the stimulation setting applied in this trial is performed for a long time, it will not be clear from this trial how the visual function changes and how long the effect will be sustained if electric stimulation is stopped. A study with a larger cohort and longer period is needed to confirm the results of this trial.

Second, we have not included advanced and very early cases. End-stage cases in which the ellipsoid zone of the fovea had totally disappeared, and early-stage cases where the visual acuity had not yet been decreased or where the sensitivity decrease was not present were not included in this study. Therefore, we cannot decide the effect of TdES on cases with advanced disease condition or cases without central vision disorder from the results. However, we suggest that the effect of electrical stimulation on the retina with advanced retinal outer layer alteration is slight. In addition, it will be difficult to judge the effect of treatment in the early cases in which visual acuity and visual field still have not been depressed.

Third, we did not perform objective examinations such as electroretinography.

Although the previous report of TES for RP patients has shown significant improvements of the ERGs and demonstrated improvement of retinal function by electrical stimulation, all of the evaluations in our study were subjective tests.

Fourth, detailed genetic investigations have not been done for our cohorts. It is undeniable that the effect of electrical stimulation varies depending on the type of genetic abnormality. There are few reports of TES on patients whose genotype was determined^19^. Further investigation about the effects of TdES on RP patients with different genotypes is needed.

Fifth, the phosphene threshold was not measured accurately. The electrically-evoked phosphene threshold is used as index of residual retinal function in the study of retinal prostheses^20^. Therefore, it is unclear whether there is a significant correlation between residual retinal function presumed from the phosphene threshold and the effect after TdES.

It was reported that the amplitudes of the ERGs were smaller when waveforms are picked-up with skin electrodes than that of corneal electrodes^21,22^. However, TdES is done after confirming that phosphene was preceived by all cases. Therefore, it is considered that the effect of the electrical stimulation using skin electrode was obtained similarly to that using the corneal electrode.

In conclusion, TdES using our procotocol does not cause any complications. The BCVA and MD value of HFA10-2 were significantly improved after TdES. We conclude that TdES with skin electrode is a safe therapeutic option for patients with RP. This trial may be the prototype trial using TdES for RP patients and contribute to the development of safe and easy method of retinal and optic nerve disease by conduction further long-term research.

## Methods

This clinical trial was an investigator-led, prospective, non-randomized, open-label, uncontrolled trial conducted at the Chiba University Hospital in Japan. This was a 12 weeks trial consisting of 6 TdES treatments on patients with RP. The TdES was applied every 2 weeks for 6 weeks. This trial was registered with the UMIN Clinical Trials Registry, UMIN000028190. The date of registration was July 12, 2017.

The diagnosis of RP was made by a history of a progressive increase in the degree of night blindness, visual field constriction, photophobia, reduced or absence of the electroretinograms (ERGs), and ophthalmoscopic findings, e.g., attenuated retinal vessels, bone-spicule-like pigment clumping, and optic disc pallor. Only typical RP patients were studied. Patients with central RP, retinitis punctata albescens, RP sine pigmento, and sectorial RP were excluded.

**Figure 3:**
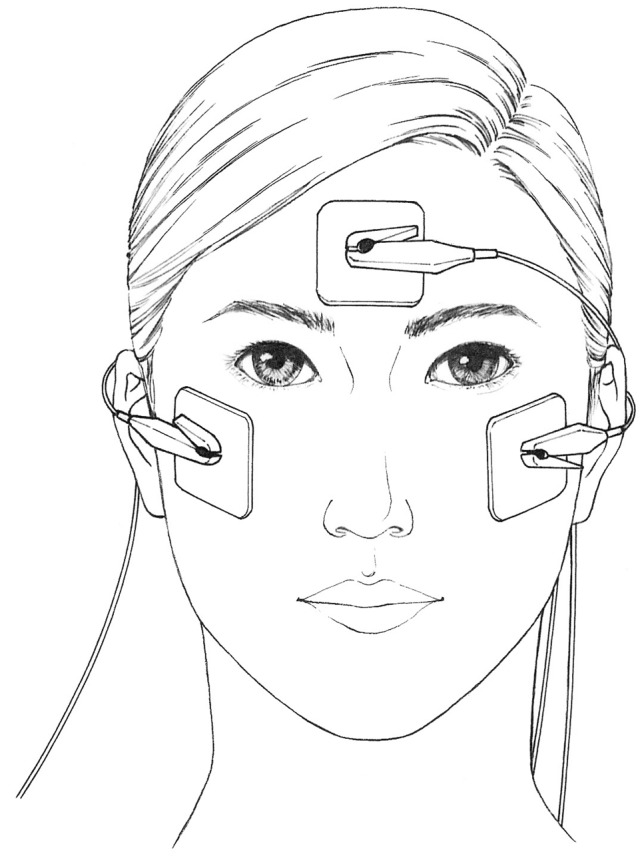
Positions of skin electrodes. Electrodes are applied to the skin at the center of the forehead of the patient and on the lower eyelid’s ear side of both eyes.

The patients underwent TdES consisting of 10-ms biphasic pulses at 20 Hz for 30 minutes. All patients were stimulated bilaterally with 1.0 mA simultaneously at the Chiba University Hospital by physicians. The electrical pulses were obtained from a prototype equipment jointly developed with Mayo Co., Ltd. (Aichi, Japan). One electrode was placed on the lower eyelid lateral to the midline of both eyes, and the other patched at the center of the forehead (Fig. 3).

### Eligibility criteria

Eligible patients are those who meet all of the following inclusion criteria and who do not have any listed exclusion criteria.

#### Inclusion criteria

1. Clinically diagnosed with typical retinal pigmentosa and age ≥20-years and ≤80-years.
2. HFA 10-2 performed twice within 6 months with reliability fixation defective rate <20%, false positive rate <15%, false negative rate <33%, and difference in the mean center four points of retinal sensitivity within 5 dB and both values were less than 30 dB.
3. Decimal visual acuity from 0.1 to 0.7.
4. Concentric constriction of the visual field to 10 degrees for target I/4 of Goldmann perimetry.
5. HFA 10-2 MD values up to −10 dB.
6. Patients who signed consent document with sufficient understanding after receiving explanation for the responsibilities of participating in this trial.
7. Regular hospital visits every 2 weeks for 3 months.

#### Exclusion criteria

1. Presence of macular lesions such as vitreous macular traction syndrome, macular edema, epiretinal membrane, and myopia with posterior staphyloma
2. Having undergone intraocular surgery within 3 months of the beginning of this trial.
3. Patients who changed the dose and usage of isopropyl unoprostone^23^, calcium antagonist, and helenien within 31 days before the screening examination.
4. History of allergy to mydriatic agents and eye surface anesthetic.
5. Diagnosed with diabetic retinopathy.
6. Presence of conjunctival inflammation, infection, or severe dry eye.
7. Patients who were pregnant, breastfeeding, may be or planned to be pregnant during the trial period.
8. History of optic nerve disease.
9. Presence of grade 3 or higher Emery-Little grade^24^ cataract or posterior capsule opacification which affected the visual function.
10. MD value of HFA 10-2 has deteriorated by 3 dB or more per year.
11. Patients who had not experienced any deterioration in the visual acuity, OCT findings, GP findings, and HFA 10-2 visual field sensitivities in the last 3 years.
12. Patients participating in other clinical trials.
13. Patients under investigational responsibility (shared) doctors judged inappropriate for participation in this trial.

The primary endpoint was safety, and the secondary endpoints were the changes in the BCVA, Humphrey field analyzer (HFA) 10-2, Goldmann perimetry (GP) and answers to visual function questionnaire-25 (VFQ-25).

Electrical stimulation was performed every 2 weeks for 6 sessions, and the patients were assessed before beginning the TdES (baseline), 1 hour after the end of treatment, and at 2 weeks after the TdES.

The TdES and examination schedule of this trial is shown in Table 2. This study protocol was approved by the Institutional Review Board of Chiba University Hospital. This trial was declared and registered at the Pharmaceuticals and Medical Devices Agency in May 26, 2017. A written informed consent was obtained from all patients before enrollment.

**Table 2:** Treatment and examination schedule.

	Screening/baseline	Treatment												Follow-up	Drop out
		0 week		2 week		4 week		6 week		8 week		10 week		12 week	
		before	after	Before	after	before	after	before	after	before	after	before	after		
	Visit 1–2	Visit 3		Visit 4		Visit 5		Visit 6		Visit 7		Visit 8		Visit 9	
IC	**○**														
TdES		①		②		③		④		⑤		⑥			
BP	**○**	**○**	**○**	**○**	**○**	**○**	**○**	**○**	**○**	**○**	**○**	**○**	**○**	**○**	**○**
VA	**○**	**○**	**○**	**○**	**○**	**○**	**○**	**○**	**○**	**○**	**○**	**○**	**○**	**○**	**○**
HFA	**○**						**○**						**○**	**○**	**○**
GP	**○**													**○**	**○**
OCT	**○**													**○**	**○**
VFQ	**○**						**○**						**○**	**○**	**○**
Slit	**○**						**○**						**○**	**○**	**○**
IOP/fds	**○**													**○**	**○**
AEs	**○**														

Abbreviations: I.C.: informed consent, B.P.: Blood Pressure, Slit: slit lamp examination, I.O.P.: Intraocular pressure.

All patients had a complete ophthalmic examination including measurements of the BCVA and the intraocular pressure (IOP). In addition, slit-lamp examinations and indirect ophthalmoscopy were performed. The BCVA was measured monocularly with a Japanese standard Landolt ring chart (System Charts SC-2000 Nidek Instruments, Gamagori, Japan) at 5 m. The decimal visual acuities were converted to the logarithm of the minimum angle of resolution (logMAR) units for the statistical analyses. The BCVA was also assessed with the Early Treatment of Diabetic Retinopathy Study chart (ETDRS; CSV-1000LanC VectorVision, Ohio, USA) at a distance of 2.5 m. The luminance for the tests was 85 cd/m2 which is the luminance recommended for vision testing by the USA National Academy of Sciences and adopted by the FDA as the required testing light level for clinical trials.

The mean deviation of retinal sensitivity was determined with the Humphrey Visual Field Analyzer III (Model 850; Carl Zeiss Meditec, Inc., Dublin, CA, USA) using the Swedish Interactive Threshold Algorithm Standard 10-2 protocol. The mean sensitivity of the central 4 points determined by HFA10-2 were also averaged and analyzed. The area of the visual field with target I/4 of GP were determined with the ImageJ software.

Patients answered the Japanese version of National Eye Institute (NEI) VFQ-25^25^ to evaluate subjective symptoms and quality of life. The NEI VFQ-25 is made up of 25 questions that addressed 12 aspects of daily living: general health, general vision, near vision, distance vision, driving, peripheral vision, colour vision, ocular pain, role limitation, dependency, social function, and mental health. In this study, compo 9 which comprehensively evaluates nine aspects (the general vision, near vision, distance vision, peripheral vision, colour vision, role limitation, dependency, social function, mental health) were analyzed.

In addition, the changes of the BCVA in logMAR units, the mean deviation of HFA 10-2, and the mean sensitivities of the central 4 points of HFA 10-2 over the past 5 years were analyzed. These methods were carried out in accordance with the relevant protocol.

### Statistical analysis

The median and ranges of the continuous variables are presented, and frequencies and proportions of the categorical variables are presented. The primary endpoint and secondary endpoint were analyzed in the full analysis set, defined as all randomly assigned patients who received at least one TdES and had at least one efficacy assessment. For the efficacy analysis, the mixed effects model for repeated measures (MMRM) which can address all available post-baseline data were used to determine if the differences of the data from baseline were statistically significant. The results are presented as the mean and mean difference from the baseline, 95% confidence intervals (CIs), and P-values. Two-sided P-values of <0.05 were considered statistically significant, and all analyses were performed with the SAS software Version 9.4 for Windows (SAS Institute Inc., Cary, NC, USA).

Mayo Co., Ltd. had no role in study design, data collection, data analysis, data interpretation, or writing of this report. All authors had full access to all the data in this trial and the corresponding author had final responsibility for the decision to submit for publication.

### Statement of ethics

An informed consent was obtained from all patient for the electrical stimulation and the other examinations. The procedures used in this study conformed to the tenets of the Declaration of Helsinki. The protocol was approved by the Institutional Review Board of Chiba university hospital. This trial was notified and registered at the Pharmaceutical and Medical Devices Agency, Japan in May 25, 2017. This trial is registered with the UMIN Clinical Trials Registry, UMIN000028190 in July 12, 2017.

## Data Availability

The datasets generated during and/or analyzed during the current study are available from the corresponding author on reasonable request.
